# Accuracy of the Whooley questions and the Edinburgh Postnatal Depression Scale in identifying depression and other mental disorders in early pregnancy

**DOI:** 10.1192/bjp.2017.9

**Published:** 2018-01

**Authors:** Louise Michele Howard, Elizabeth G. Ryan, Kylee Trevillion, Fraser Anderson, Debra Bick, Amanda Bye, Sarah Byford, Sheila O'Connor, Polly Sands, Jill Demilew, Jeannette Milgrom, Andrew Pickles

**Affiliations:** 1 Section of Women's Mental Health, Institute of Psychiatry, Psychology and Neuroscience and Women's Health Academic Centre, King's College London, London, and South London and Maudsley NHS Foundation Trust, London; 2 Biostatistics and Health Informatics Department, Institute of Psychiatry, Psychology and Neuroscience, King's College London, London; 3 Section of Women's Mental Health, Institute of Psychiatry, Psychology and Neuroscience, King's College London, London; 4 Women's Health Academic Centre and Florence Nightingale Faculty of Nursing and Midwifery, King's College London, London; 5 Section of Women's Mental Health, Institute of Psychiatry, Psychology and Neuroscience, King's College London, London; 6 King's Health Economics, Institute of Psychiatry, Psychology and Neuroscience, King's College London, London; 7 Section of Women's Mental Health, Institute of Psychiatry, Psychology and Neuroscience, King's College London, London; 8 Women's Health Academic Centre, King's College London, London and Women's Health, King's College Hospital NHS Foundation Trust, London; 9 Parent-Infant Research Institute (PIRI), Austin Health, Melbourne School of Psychological Sciences, University of Melbourne, Australia; 10 Biostatistics and Health Informatics Department, Institute of Psychiatry, Psychology and Neuroscience, King's College London, London

## Abstract

**Background:**

There is limited evidence on the prevalence and identification of antenatal mental disorders.

**Aims:**

To investigate the prevalence of mental disorders in early pregnancy and the diagnostic accuracy of depression-screening (Whooley) questions compared with the Edinburgh Postnatal Depression Scale (EPDS), against the Structured Clinical Interview DSM-IV-TR.

**Method:**

Cross-sectional survey of women responding to Whooley questions asked at their first antenatal appointment. Women responding positively and a random sample of women responding negatively were invited to participate.

**Results:**

Population prevalence was 27% (95% CI 22–32): 11% (95% CI 8–14) depression; 15% (95% CI 11–19) anxiety disorders; 2% (95% CI 1–4) obsessive–compulsive disorder; 0.8% (95% CI 0–1) post-traumatic stress disorder; 2% (95% CI 0.4–3) eating disorders; 0.3% (95% CI 0.1–1) bipolar disorder I, 0.3% (95% CI 0.1–1%) bipolar disorder II; 0.7% (95% CI 0–1) borderline personality disorder. For identification of depression, likelihood ratios were 8.2 (Whooley) and 9.8 (EPDS). Diagnostic accuracy was similar in identifying any disorder (likelihood ratios 5.8 and 6).

**Conclusions:**

Endorsement of Whooley questions in pregnancy indicates the need for a clinical assessment of diagnosis and could be implemented when maternity professionals have been appropriately trained on how to ask the questions sensitively, in settings where a clear referral and care pathway is available.

**Declaration of interest:**

L.M.H. chaired the National Institute for Health and Care Excellence CG192 guidelines development group on antenatal and postnatal mental health in 2012–2014.

Mental disorders during pregnancy are common^1^ and are associated with adverse outcomes for women, pregnancy, the fetus, infant, childhood and adolescence.^2^^–^^5^ Recent clinical guidance^6^ highlights the importance of identifying depression and other mental disorders early in pregnancy, and subsequently throughout the perinatal period, to facilitate early treatment and thus potentially mitigate subsequent adverse outcomes. The emerging evidence on the prevalence and impact of perinatal mental disorders across the diagnostic spectrum suggests that the ideal tool for case identification would indicate whether depression and other mental disorders may be present. Most research has focused on identification of perinatal depression, with a recent evidence review^7^ recommending the Edinburgh Postnatal Depression Scale (EPDS),^8^ based on sensitivities of around 0.8 and specificities of 0.87, although values varied depending on the characteristics (for example ethnicity, socioeconomic status) of the study population. No studies in this review, or subsequently to our knowledge, have systematically investigated the impact of these covariates on the diagnostic accuracy of the EPDS. The review^7^ also examined the accuracy of the PHQ-2, a two-item scale, rated using a Likert scale, or a yes/no response^9^ (the latter sometimes known as the Whooley questions). Evidence on the usefulness of the Whooley questions as used in clinical practice was limited.^8^^,^^9^ Only one study^10^ recruited women in early pregnancy and examined the PHQ-2, reporting an optimal cut-point of 4 (specificity 0.79, sensitivity of 0.62) in a cohort of 213 women (13 of whom met criteria for major depressive disorder) recruited via advertisements in obstetric clinics.^10^ No data were available on use of these questions in the simpler yes/no binary format in early pregnancy. A systematic review of the Whooley questions across other settings (for men and women) reported a pooled sensitivity of 0.95 (95% CI 0.88–0.97) and pooled specificity of 0.65 (95% CI 0.55–0.74).^11^ The only study of pregnant women in this review was a study of 126 women attending a UK maternity clinic at around 26–28 weeks’ gestation who were given a self-administered questionnaire that included the Whooley questions and reported a prevalence of (minor and major) depression of 13.5% (95% CI 8.3–21); sensitivity was 100%.^12^ In view of the limited evidence on the diagnostic accuracy of available tools as used in clinical practice, we aimed to investigate the diagnostic accuracy of the Whooley questions at the first antenatal appointment (‘booking’ appointment) in identifying (a) current depressive disorder, and (b) any disorder, compared with the EPDS, using a ‘gold standard’ diagnostic instrument (the Structured Clinical Interview DSM-IV (SCID)^13^). We also aimed to examine the impact of relevant covariates on the performance of the EPDS instrument and to estimate the prevalence of mental disorders at antenatal booking.

## Method

### Study design

This was a cross-sectional survey using a sampling design stratified according to being positive or negative (saying yes or no respectively) on either Whooley questions (‘During the past month have you often been bothered by feeling down, depressed, or hopeless?’; ‘During the past month have you often been bothered by having little interest or pleasure in doing things?’), inviting a random sample of Whooley negative (W–) and all Whooley positive (W+) women to participate.

### Study setting and population

We recruited women attending their booking appointment at an inner-city maternity service in South-east London. Exclusion criteria were age <16, no response to the Whooley questions recorded, a previous comprehensive maternity booking elsewhere in the UK and a termination or miscarriage between the booking appointment and research interview.

### Study procedures

A study advertisement was included in the pre-booking information pack sent to all women in advance of their appointment. Online audit-trailed randomisation for enrolment into the study was carried out by trained researchers (research midwives and postgraduate research psychologists), once Whooley status (‘positive’/‘negative’) had been recorded by midwives. To enhance recruitment, researchers worked evenings and weekends to fit in with women's work and childcare commitments. Recruitment and data collection were carried out once Whooley status (the index test) was determined, and the reference test (the ‘gold standard’ diagnostic interview) was performed in those who consented to participate in the study. Usual care was delivered, which could include a referral to primary or secondary mental healthcare services or a request for the primary care doctor to assess the woman's mental health and whether she needs referral for treatment, depending on other aspects of the assessment carried out by the midwives.

Women who were W– were randomly selected – initially 1:4 and then 1:6 (see sample size calculation below). All potentially eligible W+ women and the randomly selected W– women were approached by a researcher (either on the day or, if not seen then, contacted by mobile telephone/email/letter), who explained the study and invited women to consider participation. Interpreters were used to explain the study and translate where needed. Researchers interviewed women within a maximum of 3 weeks of their antenatal booking appointment i.e. before any referrals for treatment made by midwives that would have had a significant impact on mental health. Written informed consent was obtained.

### Measures

The Whooley questions and the response to the additional ‘help’ question (asked in women who respond yes to the questions, to identify those women who feel they want help), and sociodemographic data are recorded by the maternity staff. At interview the following instruments were administered by researchers:

(a) EPDS,^8^ a ten-item self-administered tool, administered by iPad where available and preferred (*n* = 95), or pen and paper, using relevant language-specific tool
(b) SCID-I-Research Version^13^ Axis I mood episodes, mood disorders and anxiety disorders module; SCID Axis I eating disorders module (SCID-I) and SCID-II personality disorders subsection module for borderline personality disorder.^14^

Researchers were trained to use the SCID, a ‘gold standard’ diagnostic interview, over a 3-month period, and then met weekly with L.M.H. to achieve consensus on diagnosis.

Clinical information and Whooley status was available to these consensus meetings, although the two-item responses as responded to when they are asked within the SCID (rather than the original response to the midwives) were used when assessing diagnostic criteria. Diagnosis of major depressive disorder included mild/moderate/severe depressive episode and mixed anxiety depression; women with bipolar disorder were classified as having current bipolar disorder (no women who were diagnosed with bipolar disorder were experiencing a depressive episode).

All women were offered information about sources of help and support (for example. domestic violence, smoking and substance misuse services). Where the diagnostic interview identified a woman as having a mental disorder their midwife was informed, if the woman consented to this. Detailed standard operating procedures were used for this and other related studies^15^ including contacting a senior clinician for concerns about safety, child protection and other potential sources of harm, and when to potentially breach confidentiality; in practice no breaches of confidentiality were necessary.

### Sample size calculation

Power calculation for the two-phase design was undertaken using simulation with bootstrap estimation of confidence intervals for the weighted estimators of sensitivity, specificity and prevalence that corrected for the sample stratification. We assumed an overall prevalence of 9% depression and Whooley sensitivity of 0.95 and specificity of 0.89. Screening 6000 women by midwives, 66% of whom consent to participate, and sampling 54% of the W+ women (i.e. *n* = 400) and 6% of the W– (i.e. *n* = 200), would provide 600 women for interview; we expected 185 to be depressed. Assuming a sensitivity of 0.80 and specificity of 0.71, the width of the 95% confidence interval for the EPDS sensitivity would be 0.19 and that for specificity 0.13. A conservative estimate of power based on the 185 disease ‘cases’ only would have >90% power for a 0.8 *v.* 0.65 sensitivity and specificity difference (comparing Whooley and the EPDS). As data collection was monitored, it became clear that adjustments to sampling fractions were necessary as there were fewer W+ women being recruited than anticipated whereas the original recruitment target of 200 W– women had been reached. After discussion with the independent Data Monitoring and Ethics Committee, we then aimed for 300 W+ and 300 W– women so that the two arms were recruited over the same time period, with random sampling of W– of 1:6.

### Statistical analysis

**Fig. 1:**
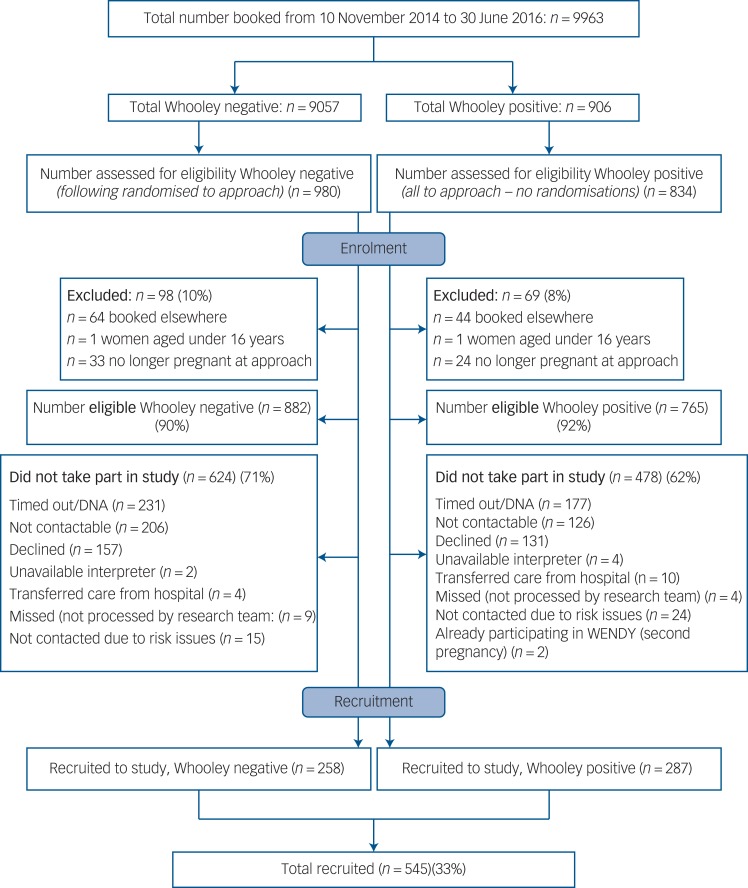
Flow chart of women through the study. DNA, did not attend.

In all analyses (apart from examining differences in sociodemographic variables between W+ and W– women), sampling weights were used to account for the bias induced by the stratified sampling. Weights were based on the number of W+ and W– women in the study, out of all those that had maternity appointment bookings at the maternity unit during the study period (the sampling frame) (Fig. 1); the weights were 906/287 for W+ and 9057/258 for W–.

DNA, did not attend.

As pre-specified in our original grant application, for both the Whooley and the EPDS, we ascertained the rates of ‘true’ and ‘false’ positives and ‘true’ and ‘false’ negatives for: (a) depressive symptoms and (b) symptoms of any mental disorder. Sensitivity, specificity, positive predictive values (PPV), negative predictive values (NPV) and likelihood ratios were calculated. Appropriate cut-off scores for the EPDS were identified using receiver operating characteristics (ROC) analysis. This was implemented using the approach outlined by Pepe^16^^,^^17^ and the *rocreg* command in Stata (v14.0). The optimal cut-off point to discriminate between states (for example depressed and not depressed) was chosen based on sensitivity, specificity, PPV and NPV. We also estimated prevalence rates of disorders based on the weighted diagnostic interview responses. Bootstrap re-sampling of the weighted estimators was used for calculation of confidence intervals (other than for the prevalences of each of the categories in the severity of SCID depression (an ordinal outcome) for which we used the confidence interval estimates generated from Stata's svy command).

As some covariates could affect the inherent discriminatory accuracy of the EPDS, we explored the effect of incorporating sociodemographic variables into the ROC curves for the EPDS using the approach described by Janes *et al*.^18^ It was assumed that the covariates affected the ROC curve only, and not the distribution among controls, and so the *rocreg* command was used in Stata (v14.0) with the *roccov* option.

### Missing data

Data for the help question were missing for six women who had responded yes to one of the two Whooley questions. Five women had completely missing data for EPDS items and were not included in EPDS analyses. Eleven women had 1–3 EPDS items missing. In total, 521 (96%) women answered questions from all SCID modules but 21 (3.9%) declined the post-traumatic stress disorder (PTSD) module (8 of whom had disclosed trauma during the interview) and other isolated non-completed modules occurred. A single round of predictive mean matching was performed using the *mi impute* function (predictive mean matching option) in Stata (v14.0) to impute missing EPDS data for the 11 women who had 1–3 items (10–30%) missing. No imputation was performed for women who had more than 30% data missing in the EPDS items, which were treated as missing observations in relevant analyses (list-wise deletion performed in Stata). To account for missing observations in the SCID items, we used inverse probability weights that accounted for the Whooley sampling, as well as variables that were significant in predicting missingness of SCID responses (EPDS total score, ethnicity and employment status). Ethical approval: the research was approved by the National Research Ethics Service, London Committee – Camberwell St Giles (ref no 14/LO/0075).

## Results

### Study sample

Between 10 November 2014 and 30 June 2016, 10 004 women attended their initial antenatal appointment at the study site; 41 did not have a Whooley response recorded so the base population consisted of 9963 women. This base population was similar to the study population for age, ethnicity and number of children (online Table DS1 available at https://doi.org/10.1192/bjp.2017.9). Of the 882 Whooley negative (W–) women that were eligible, 624 (71%) did not participate and 478 (62%) of the 765 eligible Whooley positive (W+) women did not participate (Fig. 1). Significant differences were found between the 287 W+ and the 258 W– participants, with W+ women more commonly being younger, single, living alone, having no formal educational qualifications/only high school qualifications, insecure immigration status and lower income (online Table DS2).

Using weighted estimation, the population prevalence of a SCID disorder was 27% (95% CI 22–32); with a prevalence of 11% (95% CI 8–14) for depressive disorder (of which over half were mild depressive disorder) (Table 1), 15% (95% CI 11–19) anxiety disorder, 2% (95% CI 1–4) obsessive–compulsive disorder, 0.8% (95% CI 0–1) PTSD, 2% (95% CI 0.4–3) eating disorder, 0.3% (95% CI 0.1–1) bipolar disorder I, 0.3% (95% CI 0.1–1%) bipolar disorder II; and 0.7% (95% CI 0–1) borderline personality disorder (see also Table 1).

**Table 1:** Population prevalence of diagnoses by Whooley and Edinburgh Postnatal Depression Scale (EPDS) status

	Rates, % (95% CI)				
	Whooley, positive	Whooley, negative	EPDS,^b^ positive	EPDS,^b^ negative	Prevalence
No depression	56 (49–61)	93 (89–96)	48 (34–62)	95 (93–97)	90 (86–92)
Major depression^a^	47 (40–53)	7 (4–10)	53 (37–71)	5 (3–7)	11 (8–14)
Mild depression	23 (18–29)	4 (2–7)	27 (16–41)	3 (2–6)	6 (4–9)
Moderate depression	20 (16–25)	3 (1–6)	25 (15–38)	2 (0.6–4)	4 (3–8)
Severe depression	1 (0.4–4)	0	1 (0.3–3)	0	0.1 (0–0.3)
Mixed anxiety/depression	4 (2–6)	0	1 (0–2)	0.3 (0.1–0.4)	0.4 (0.2–0.6)
Any anxiety disorder	30 (24–36)	13 (9–18)	23 (12–35)	14 (10–18)	15 (11–19)
Obsessive–compulsive disorder	5 (3–7)	2 (0.4–4)	3 (1–5)	2 (1–4)	2 (1–4)
Eating disorder	5 (2–8)	1 (0–4)	3 (1–5)	1 (0.1–3)	2 (0.4–3)
Post-traumatic stress disorder	5 (2–7)	0.4 (0.1–3)	6 (0–11)	0.1 (0–0.3)	0.8 (0–1)
Bipolar disorder I	0.3 (0.1–1)	0	0	0.04 (0–0.1)	0.03 (0–0.2)
Bipolar disorder II	0.3 (0.1–1)	0	0	0.04 (0–0.1)	0.03 (0–0.2)
Borderline personality disorder	4 (2–6)	0.4 (0–3)	2 (1–4)	0.5 (0–1.3)	0.7 (0–1)
Any SCID	67 (60–73)	22 (17–29)	67 (51–84)	21 (16–27)	27 (22–32)

SCID, Structured Clinical Interview DSM-IV.

a. This includes major depressive disorder and mixed anxiety and depression. Minor depression rates were 10% (95% CI 7–14) in Whooley positive; 2% (95% CI 0.8–5) in Whooley negative; 9% (95% CI 4–22) in EPDS positive; 2% (95% CI 0.8–4) in Whooley negative; an overall prevalence of 3% (95% CI 1 to 5%).

b. Cut-off of 12/13 used for EPDS negative/positive.

No adverse events occurred from being asked the Whooley questions or taking part in the research interview. Health professionals were informed when severe disorders were identified and all participants consented to this information being shared with their midwife and/or general practitioner.

### Diagnostic accuracy of the Whooley questions for depression

SCID depression was found in 17 (6.6%) W– and 130 (45.3%) W+ women, where W+ was defined as answering yes to either one of the two questions. After adjustment for weighting, SCID depression was estimated to occur in 597 (7.6%) W– and 410 (45.3%) W+ women; no depression was found in 8460 (93.4%) W– and 496 (54.7%) W+. Weighted sensitivity was 0.41, specificity 0.95, PPV 0.45, NPV 0.93, likelihood ratio (positive) 8.2, likelihood ratio (negative) 0.62 and area under the curve (AUC) for ROC curve 0.37 (95% CI 0.34–0.40). For W+ defined as answering yes to either one of the two Whooley questions and yes to the additional ‘help’ question, sensitivity was 0.08 and specificity 0.99, with a PPV 0.66, NPV 0.83, likelihood ratio (positive) 8, likelihood ratio (negative) 0.93 and AUC for ROC curve 0.21 (95% CI 0.19 to 0.23). When W+ was defined as answering yes to both of the two Whooley questions, SCID depression was found in 67 (16.2%) W− and 80 (60.6%) W+ women. Weighted sensitivity was 0.25, specificity 0.98, with a PPV 0.61, NPV 0.84, likelihood ratio (positive) 12.5, likelihood ratio (negative) 0.77, and AUC of the ROC curve 0.24 (95% CI 0.21 to 0.26).

### Diagnostic accuracy of the EPDS for depression

**Fig. 2:**
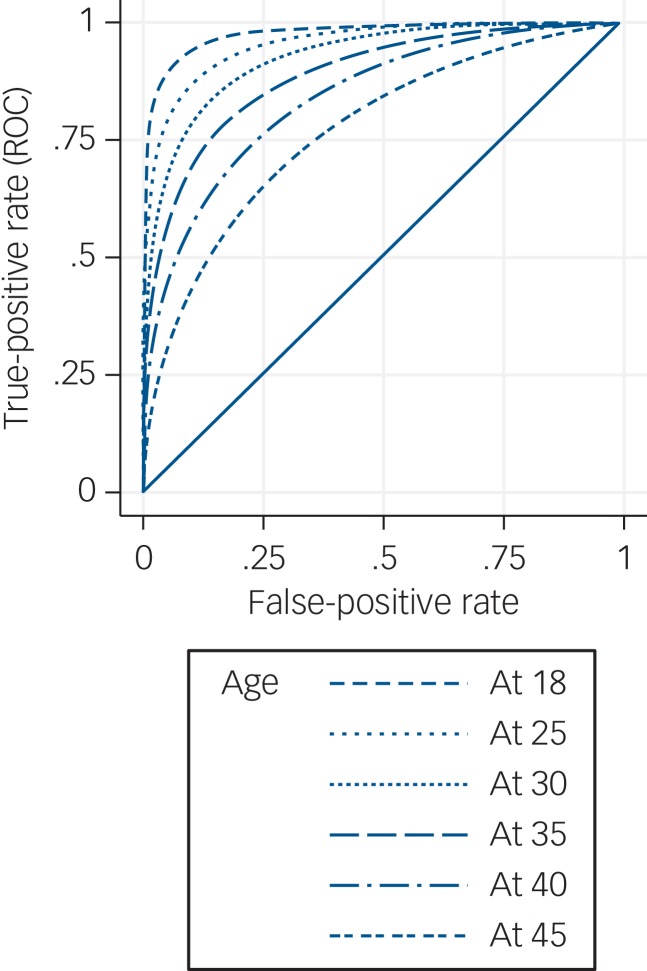
Receiver operating characteristic (ROC) curves of Edinburgh Postnatal Depression Scale with covariate adjustment.

The range of EPDS scores was 0–28, with a median of 7 (IQR 4–13). Using a cut-off of 12/13 (which was optimal for diagnostic accuracy – see Table 2), SCID depression was found in 49 (12.2%) EPDS– women and 98 (68.5%) EPDS+ women. This cut-off resulted in weighted sensitivity 0.59, specificity 0.94, PPV 0.52, NPV 0.95, likelihood ratio (positive) 9.8, likelihood ratio (negative) 0.44, and AUC for the ROC curve of 0.89 (95% CI 0.88–0.90) (see online Table DS3 for 2 × 2 tables of weighted prevalences). ROC curve analysis found no evidence of a difference in the ability of the EPDS to discriminate between ‘cases’ and ‘non-cases’ among the five ethnicity categories (χ^2^(4) = 3.52, *P* = 0.48), the five income categories (χ^2^(4) = 6.89, *P* = 0.14), the three education categories (χ^2^(2) = 2.48, *P* = 0.29) nor for those who used an iPad rather than paper for completion (χ^2^(1) = 1.48, *P* = 0.22). However, there was evidence that the discriminatory ability of the EPDS decreased with increasing age analysed as a continuous variable (χ^2^(1) = 19.12, *P* < 0.0001) (Fig. 2). The EPDS also performed better in those who participated in the study using an interpreter (*n* = 40) (χ^2^(1) = 5.45, *P* = 0.02).

**Table 2:** Performance of Edinburgh Postnatal Depression Scale for different cut-off values for depression

Cut-off	Sensitivity	Specificity	Positive predictive value	Negative predictive value
11	0.73	0.88	0.40	0.97
12	0.68	0.92	0.48	0.96
13	0.59	0.94	0.52	0.95
14	0.46	0.95	0.50	0.94
15	0.44	0.96	0.56	0.94

### Diagnostic accuracy for any disorder

A SCID disorder was found in 242 (45%) participants (27% had depressive disorders and 21% had anxiety disorders) and 294 (54%) had no SCID diagnosis. Using weighted estimation, the population prevalence of any disorder was 27% (95% CI 22–32).

#### Whooley questions

A SCID disorder was found in 55 (21.9%) W– women and 187 (65.6%) W+ women, where W+ was defined as answering yes to either one of the two questions. Weighted sensitivity (online Table DS4 for 2 × 2 table of weighted prevalences) was 0.23, specificity 0.96, PPV 0.66, NPV 0.78, likelihood ratio (positive) 5.8, likelihood ratio (negative) 0.80 and AUC for ROC curve 0.21 (95% CI 0.2–0.23). When the ‘help’ question was added, sensitivity was 0.05, specificity 1.00, PPV 0.86, NPV 0.65, negative likelihood ratio 0.95 and AUC for ROC curve was 0.11 (95% CI 0.10 to 0.12). When W+ was defined as answering yes to both questions the sensitivity was 0.06, specificity 0.99, PPV 0.78, NPV 0.66, likelihood ratio (positive) 6, likelihood ratio (negative) 0.95 and AUC for the ROC curve was 0.12 (95% CI 0.11–0.13).

### EPDS

Using a cut-off of 12/13, 121 (30.7%) EPDS– women and 121 (85.2%) EPDS+ women had a SCID disorder (online Table DS4 for 2 × 2 table of weighted prevalences). Weighted sensitivity was 0.3, specificity 0.95, PPV 0.67, NPV 0.79, likelihood ratio (positive) 6, likelihood ratio 0.74 (negative) and AUC for ROC curve 0.74 (95% CI 0.73–0.75).

## Discussion

### Main findings

The ten-item EPDS performed better in correctly identifying major depression (likelihood ratio 9.8) than the two-item Whooley questions (likelihood ratio 8.2) in early pregnancy. However, the difference in diagnostic accuracy was not large, and both tools had high specificity. There have been no comparable studies in early pregnancy but our study, which aimed to validate the use of the Whooley questions when routinely asked face-to-face by midwives, suggests that the Whooley has a lower sensitivity for identification of depression than often reported in other studies. This may be because of different methods of administration of the questions, as previous studies have not usually validated the Whooley as used in maternity practice, but rather have used a written format administered by a researcher.^11^ However, the low sensitivity of the Whooley may also partly be because of the variation in how the questions were asked in clinical practice by staff who had not usually been trained in perinatal mental health. The sensitivity of the EPDS was comparable with some studies although others have reported higher sensitivity in pregnancy.^7^ The (generally) lower sensitivity of the screening tools found in our study compared with others, may also reflect the larger, more representative study population included here (which in this study included women from very diverse backgrounds and those who did not speak English) and, for the Whooley, the delay between the midwives asking the questions and the diagnostic interview being administered.

The administration of the ten questions of the EPDS is, however, potentially burdensome in busy maternity settings and may outweigh the slightly improved diagnostic accuracy of the EPDS when considering how to identify antenatal depression in routine maternity care. It is therefore useful to note that use of iPads in administering the EPDS did not reduce its effectiveness. The EPDS, a self-complete tool, therefore could be completed by women when they are not being seen by a midwife for example while in the waiting room. It should be noted though, that we found that responses to the EPDS in older women were less discriminatory in identifying depression. We speculate that this may reflect a longer duration of mental disorders and associated anticipated discrimination^19^ or self-perceived resilience.

In clinical practice, maternity professionals need to identify whether or not a woman has any mental disorder, not only depression. It is therefore particularly important that, in this study, there was little difference in diagnostic accuracy between the Whooley questions and the EPDS in identifying a mental disorder: both tools had low sensitivity (0.23 for the Whooley, 0.3 for the EPDS) and high specificity (0.94 and 0.95, respectively), with similar likelihood ratios. In practice, this means that pregnant women presenting for their first antenatal appointment who have a mental disorder are 5.8 times more likely to say yes to one of the Whooley questions, (or six times more likely to score above 12 on the EPDS) than those without a mental disorder, supporting use of either instrument in routine practice; a positive screen then needs to be followed by a clinical assessment by an appropriate health practitioner to establish the clinical diagnosis and appropriate intervention.^20^

### Implications

Our findings confirm that the Whooley questions are a useful tool for case identification in early pregnancy in settings where face-to-face questions can be asked as part of a general discussion about health; a positive Whooley response suggests the respondent may have a mental disorder (not necessarily depression), and needs further clinical assessment. Questions about mental health that can be asked quickly and easily by midwives at routine planned contacts also indicate to pregnant women that this is a service that addresses mental and physical health; such questions, in the context of a supportive open discussion, also provide an opportunity to discuss a woman's replies in the context of her psychosocial circumstances. There is evidence that how the Whooley questions are asked by midwives determines their acceptability^21^ and the Whooley questions should therefore only be implemented when midwives and obstetricians have been appropriately trained on how to ask the questions sensitively, in settings where a clear referral and care pathway is available.

This study does not provide direct evidence on whether midwives should routinely ask screening questions, as women were not randomised to routine enquiry to examine whether being asked improves health outcomes. However, there is evidence that unless mental health questions are asked routinely, women from some backgrounds (such as non-White groups) are less likely to be asked about mental health.^22^^,^^23^ Routine enquiry could therefore address ‘the inverse care law’ in relation to maternity care,^22^ and practitioners’ unconscious biases. Standardised questions are sometimes viewed as a ‘tick box’ exercise by staff and women,^24^ but it is striking that where women were asked the EPDS questions (i.e. when they needed an interpreter) there was significantly better discriminatory performance of the instrument suggesting that being asked questions face-to-face may facilitate disclosure of problems. A similar study, validating the questions when asked by relevant health professionals such as health visitors in the postpartum period, would be useful.

### Strengths and limitations

This study assessed the accuracy of the Whooley questions being asked by midwives at a routine maternity contact rather than validating responses to researchers. Other strengths include the use of a diagnostic interview; an efficient, well-powered study design; and a diverse study population. Limitations include the relatively low response rate, the delay in administering the EPDS and the SCID after the initial booking appointment when the Whooley questions were asked, some missing data and the use of a single maternity site in inner-city London.

In conclusion, the two-item Whooley questions can be asked routinely by midwives when women attend for their routine antenatal booking appointment and are a quick method for identifying that a mental disorder may be present. This study also supports an alternative strategy of a self-complete EPDS, using a tablet or paper. A positive screen will necessitate further comprehensive psychosocial assessment for identification of the type and severity of mental disorder and related problems, and subsequent treatment to reduce maternal and fetal morbidity.

## Supplementary material

For supplementary material accompanying this paper visit https://doi.org/10.1192/bjp.2017.9. click here to view supplementary material
