# Supplementary material for: Accuracy of the Whooley questions and the Edinburgh Postnatal Depression Scale in identifying depression and other mental disorders in early pregnancy
Source: Br J Psychiatry. 2018 Jan;212(1):50–6. doi: 10.1192/bjp.2017.9 (PMC6457164; doi:10.1192/bjp.2017.9)
Supplement: Supplementary file 1 [file S0007125017000095sup001.docx]

Data supplement to Howard et al. Accuracy of the Whooley questions and the Edinburgh Postnatal Depression Scale in identifying depression and other mental disorders in early pregnancy. Br J Psychiatry doi: 10.1192/bjp.bp.117.206185

**Table DS1** Characteristics of study population and base population

|  | Study population | Base population* |
| --- | --- | --- |
| *Ethnicity:*  White Black Asian Mixed Other | 284 (52%) 177 (32%) 25 (5%)  23 (4%) 36 (7%) | 4914 (51%) 3162 (33%)  594 (6%)  308 (3%)  646 (7%) |
| *Age*  <20 20-29 30-39 40+ | Mean: 32.85, range: 16-47.5  8 (1%) 150 (28%) 341 (63%)  46 (8%) | Mean: 31.67, Range: 14-52  232 (2%) 3048 (30%) 6240 (61%)  705 (7%) |
| *Other children*  None 1 >=2 | 271 (50%) 175 (32%) 99 (18%) | 5077 (50%) 3209 (31%) 1939 (19%) |

*missing data not included; base population covers period from 1.11.2014-30.6.2016

**Table DS2** Characteristics of the study population by Whooley and EPDS status

|  | **Whooley Positive** | **Whooley Negative** | ***P* value** | **EPDS* Positive** | **EPDS Negative** | ***P* value** | **Total** |
| --- | --- | --- | --- | --- | --- | --- | --- |
| ***Age*** | | | | | | | |
| <25 | 45 (16%) | 12 (5%) | P<0.001 | 26 (18%) | 31 (8%) | P=0.002 | 57 (10%) |
| 25–29 | 53 (18%) | 48 (19%) |  | 29 (20%) | 72 (18%) |  | 101 (19%) |
| 30–39 | 165 (57%) | 176 (68%) |  | 80 (56%) | 261 (65%) |  | 341 (63%) |
| 40+ | 24 (8%) | 22 (9%) |  | 8 (6%) | 38 (9%) |  | 46 (8%) |
| ***Ethnicity*** | | | | | | | |
| White | 140 (49%) | 144 (56%) | P=0.460 | 63 (44%) | 221 (55%) | P=0.156 | 284 (52%) |
| Black | 99 (34%) | 78 (30%) |  | 58 (41%) | 119 (30%) |  | 177 (32%) |
| Asian | 14 (5%) | 11 (4%) |  | 7 (5%) | 18 (5%) |  | 25 (5%) |
| Mixed | 15 (5%) | 8 (3%) |  | 5 (4%) | 18 (5%) |  | 23 (4%) |
| Other | 19 (7%) | 17 (7%) |  | 10 (7%) | 26 (6%) |  | 36 (7%) |
| ***Partner status*** | | | | | | | |
| Partner not cohabiting | 59 (21%) | 23 (9%) | P<0.001 | 29 (20%) | 53 (13%) | P<0.001 | 82 (15%) |
| Cohabiting/ married | 178 (62%) | 214 (83%) |  | 80 (56%) | 312 (78%) |  | 392(72%) |
| Single | 50 (17%) | 21 (8%) |  | 34 (24%) | 37 (9%) |  | 71 (13%) |
| ***Living status*** | | | | | | | |
| Alone | 46 (16%) | 25 (10%) | P<0.001 | 30 (21%) | 41 (10%) | P<0.001 | 71 (13%) |
| Spouse/partner | 174 (61%) | 202 (79%) |  | 79 (56%) | 297 (74%) |  | 376 (70%) |
| Parents/family | 29 (10%) | 17 (7%) |  | 13 (9%) | 33 (8%) |  | 46 (9%) |
| Friends | 10 (4%) | 4 (2%) |  | 8 (6%) | 6 (2%) |  | 14 (3%) |
| Other | 25 (9%) | 9 (4%) |  | 12 (8%) | 22 (6%) |  | 34 (6%) |
| ***Employment status*** | | | | | | | |
| Working | 175 (61%) | 180 (70%) | P=0.086 | 83 (58%) | 272 (68%) | P=0.051 | 355 (65%) |
| Student | 8 (3%) | 8 (3%) |  | 5 (4%) | 11 (3%) |  | 16 (3%) |
| Unemployed | 41 (14%) | 23 (9%) |  | 19 (13%) | 45 (11%) |  | 64 (12%) |
| Homemaker | 38 (13%) | 36 (14%) |  | 19 (13%) | 55 (14%) |  | 74 (14%) |
| Not working due to illness/Other | 23 (8%) | 11 (4%) |  | 16 (11%) | 18 (4%) |  | 34 (6%) |
| ***Immigration status*** | | | | | | | |
| UK National | 168 (63%) | 162 (63%) | P=0.010 | 77 (54%) | 253 (63%) | P = 0.023 | 330 (61%) |
| EEA citizen | 39 (14%) | 38 (15%) |  | 17 (12%) | 60 (15%) |  | 77 (14%) |
| Indefinite leave to remain/  Exceptional leave to remain | 31 (11%) | 24 (9%) |  | 19 (13%) | 36 (9%) |  | 55 (10%) |
| Temporary admission/ Awaiting initial decision | 34 (12%) | 11 (4%) |  | 20 (14%) | 25 (6%) |  | 45 (8%) |
| Other | 15 (5%) | 23 (9%) |  | 10 (7%) | 28 (7%) |  | 38 (7%) |
| ***Needs an interpreter*** | | | | | | | |
| Yes | 27 (9%) | 13 (5%) | P = 0.051 | 13 (9%) | 27 (7%) | P = 0.350 | 40 (7%) |
| ***Educational history*** | | | | | | | |
| No/only school qualifications | 78 (27%) | 42 (16%) | P=0.004 | 36 (25%) | 84 (21%) | P=0.063 | 120 (22%) |
| Training/Higher Certificate/  Diploma | 77 (27%) | 66 (26%) |  | 45 (31%) | 98 (24%) |  | 143 (26%) |
| Degree/  Postgraduate | 132 (46%) | 150 (58%) |  | 62 (43%) | 220 (55%) |  | 282 (52%) |
| ***Other children*** | | | | | | | |
| None | 147 (51%) | 124 (48%) | P=0.716 | 69 (48%) | 202 (50%) | P=0.813 | 271 (50%) |
| 1 | 88 (31%) | 87 (34%) |  | 49 (34%) | 126 (31%) |  | 175 (32%) |
| ≥2 | 52 (18%) | 47 (18%) |  | 25 (17%) | 74 (18%) |  | 99 (18%) |
| ***Booked late for antenatal care*** | | | | | | | |
| No | 229 (80%) | 221 (86%) | P=0.071 | 114 (80%) | 336 (84%) | P=0.296 | 450 (83%) |
| Yes | 58 (20%) | 37 (14%) |  | 29 (20%) | 66 (16%) |  | 95 (17%) |
| ***Income*** | | | | | | | |
| < £15000 | 51 (24%) | 26 (13%) | P=0.002 | 39 (13%) | 38 (34%) | P<0.001 | 77 (19%) |
| £15,000-£30,999 | 43 (20%) | 28 (14%) |  | 47 (15%) | 24 (22%) |  | 71 (17%) |
| £31,000-£45,999 | 27 (13%) | 33 (16%) |  | 47 (15%) | 13 (12%) |  | 60 (14%) |
| £46,000-£60,999 | 29 (14%) | 34 (17%) |  | 52 (17%) | 11 (10%) |  | 63 (15%) |
| £61,000 or more | 60 (29%) | 85 (41%) |  | 120 (39%) | 25 (23%) |  | 145 (35%) |

*Cut-off of 13 is used as this was found to have a good compromise between the sensitivity and specificity.

**Table DS3** 2x2 tables of weighted population prevalences for calculation of sensitivity and specificity for depression

|  | **Whooley negative** | **Whooley positive** |
| --- | --- | --- |
| **No depression** | 8460 | 496 |
| **SCID depression diagnosis** | 597 | 410 |
|  | **EPDS* negative** | **EPDS positive** |
| **No depression** | 8398 | 557 |
| **SCID depression diagnosis** | 410 | 597 |

*Edinburgh Postnatal Depression Scale

**Table DS4** 2x2 tables of weighted population prevalences for sensitivity and specificity for any mental disorder

|  | **Whooley negative** | **Whooley positive** |
| --- | --- | --- |
| **No SCID diagnosis** | 6881 | 309 |
| **SCID diagnosis** | 1931 | 590 |
|  | **EPDS* negative** | **EPDS positive** |
| **No SCID diagnosis** | 6804 | 386 |
| **SCID diagnosis** | 1756 | 765 |

* Edinburgh Postnatal Depression Scale
